# Ecologically‐Valid Emotion Signatures Enhance Mood Disorder Diagnostics

**DOI:** 10.1002/advs.202505524

**Published:** 2026-01-05

**Authors:** Shuyue Xu, Linling Li, Ting Luo, Gan Huang, Li Zhang, Benjamin Becker, Zhen Liang

**Affiliations:** ^1^ School of Biomedical Engineering, Medical School Shenzhen University Shenzhen China; ^2^ Guangdong Provincial Key Laboratory of Biomedical Measurements and Ultrasound Imaging Shenzhen China; ^3^ State Key Laboratory of Brain and Cognitive Sciences The University of Hong Kong Hong Kong China; ^4^ Department of Psychology The University of Hong Kong Hong Kong China

**Keywords:** BD, emotion, fMRI, MDD, naturalistic

## Abstract

Mood disorders, including Major Depressive Disorder (MDD) and Bipolar Disorder (BD), are highly prevalent conditions. These disorders are characterized by persistent emotional dysregulation and substantial functional impairments. Despite extensive neuroimaging research, reliable neurofunctional markers remains elusive. To address this gap, we propose a novel approach that utilizes Divergent Emotional Functional Networks (DEFN), derived from functional magnetic resonance imaging (fMRI) in naturalistic contexts.By integrating naturalistic emotion induction, dynamic functional connectivity (dFC), and machine learning, we identified emotion‐specific functional patterns in healthy individuals with an accuracy of 83.99%. The DEFN was subsequently validated in clinical datasets, including a multi‐site MDD cohort (Hiroshima University: MDDs = 63, HCs = 111; University of Tokyo: MDDs = 62, HCs = 96) and an independently BD cohort (BDs = 59, HCs = 50). Using static functional connectivity (sFC) and nested 10‐fold cross‐validation, DEFN‐based models (MDD: 70.33%, BD: 75.18%) significantly outperformed baseline models in classifying patients and HCs (MDD: 70.33% vs. 57.58%; BD: 75.18% vs. 63.18%). Additionally, DEFN demonstrates highly reproducibility across age and sex, supporting the robustness of DEFN model. In conclusion, the DEFN approach presents a promising, reproducible, and clinically relevant neural marker for diagnosing, offering potential for more effective and timely interventions.

## Graphical abstract

This study identifies ecologically‐valid Divergent Emotional Functional Networks (DEFN), derived from dynamic functional connectivity during naturalistic movie watching. The DEFN reliably enhances diagnostic accuracy for mood disorders, including major depressive and bipolar disorders, demonstrating strong reproducibility across demographic factors and offering a promising framework for emotion‐specific neural characterization in clinical applications.

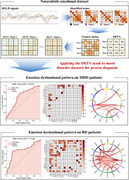

## 1 Introduction

Mood disorders are highly prevalent and debilitating conditions that have become the leading contributor to the burden of disease collaborators [1]. Major depressive (MDD) and bipolar disorder (BD) are among the most common mood disorders [2]. These disorders are characterized by persistent emotional dysregulation, including pervasive sadness, or loss of interest or pleasure in activities once enjoyed (anhedonia) [3, 4]. Both lead to significant functional impairments and decreased quality of life [2, 5]. Several studies have combined case‐control neuroimaging designs or meta‐analytic approaches to demonstrate differences in brain activity or connectivity between individuals with MDD or BD and healthy individuals [6, 7, 8, 9]. While these studies have provided valuable insights, they have failed to establish reliable and reproducible neurofunctional markers that can be used for clinical diagnosis or treatment selection [10]. While large‐scale studies have employed resting‐state functional magnetic resonance imaging (rs‐fMRI) and structural imaging to investigate diagnostic markers for depression, findings have often been inconsistent and difficult to replicate across cohorts [11]. Limitations of this top‐down approach using case‐control studies have long been discussed and have, for instance, inspired the development of the Research Domain Criteria Approach, a bottom‐up approach with a focus on the underlying neurobehavioral domains [10, 12].

One major challenge in linking behavioral dimensions to neuroimaging findings in mood disorders is the lack of emotion‐specific neural markers that can guide clinical diagnosis. Despite significant progress in the development of emotion‐specific neural signatures in affective neuroimaging using task‐based fMRI [13, 14], many of these paradigms have relied on abstract or highly controlled emotional stimuli and activation‐based analyses, which may limit their ecological validity and translational potential in real‐world contexts [15, 16]. In response, recent studies have incorporated clinically grounded instruments, such as self‐report scales embedded within neuroimaging designs, providing promising avenues for enhancing ecological and clinical relevance [17, 18]. Complementary to these approaches, naturalistic paradigms, such as movie viewing, offer high ecological validity and improved participant engagement, which could facilitate the capture of more naturalistic emotional responses. Despite progress in neuroimaging research, promising reports of clinically meaningful emotional‐dysfunction marker in these disorders were not replicated, or only partially replicated [19, 20]. This results in prolonged and ineffective treatment, poorer prognoses, and increased healthcare costs [21]. To extend current efforts, we introduce a novel two‐step framework that integrates recent progress in the development of emotion‐specific network markers under naturalistic emotion experience with network‐based diagnosis of mood disorders (Divergent Emotional Functional Networks, DEFN). By providing a more reproducible and clinically relevant neural marker, this approach has the potential to significantly enhance early‐stage diagnostic accuracy, ultimately paving the way for more effective and timely interventions.

Functional magnetic resonance imaging (fMRI) is widely used in affective and psychiatric neuroscience to determine how the brain processes emotions and dysregulation in these processes in mental disorders [13, 14, 22]. While paradigms using tasks to induce specific emotional states have been extensively utilized in healthy subjects, the vast majority of studies in patients have focused on rs‐fMRI given the easier implementation into the clinical context and reduced burden for the patients [23, 24, 25]. While some of the current limitations of rs‐fMRI relate to technical issues inherent to the technique, e.g., head motion, wakefulness, or replicability [26, 27], rs‐fMRI is limited due to the lack of specificity for specific emotional states. Recent developments have therefore proposed novel naturalistic paradigms during which individuals are exposed to movies or narratives to approximate real‐life environments and, as such, provide a more realistic reflection of brain activity during emotional experiences in ecological environments [28, 29, 30, 31]. These naturalistic paradigms offer new insights into how the human brain operates in real life, which is more dynamic and complex than the abstract tasks designed for laboratory settings. From a clinical perspective, the naturalistic paradigm shares similar advantages with the resting‐state paradigm in terms of participant compliance but imposes implicit behavioral constraints. Specifically, naturalistic paradigm fMRI significantly alleviates anxiety related to scanner performance and head movement, increases participant engagement and synchronization between subjects, allowing for more targeted research into mood disorders [32, 33].

Functional connectivity (FC) reflects the information integration and interaction between different brain regions and large‐scale networks by observing the synchrony of blood‐oxygen‐level‐dependent (BOLD) signals across these brain areas [34, 35]. FC analysis usually assumes that brain activity remains stable throughout the data acquisition of several minutes, while connectivity changes dynamically over the period. This static analysis may overlook subtle brain connectivity features that vary over time. Therefore, dynamic functional connectivity (dFC) has increasingly attracted interest in recent years, as it can detect transient brain functional connectivity patterns and reveal dynamic transitions between different brain states [36, 37, 38]. DFC, as an effective tool for exploring the integration of complex brain networks, has been widely applied in the research of mental neurological disorders, including mood disorders [39, 40, 41], schizophrenia [42, 43], Attention Deficit Hyperactivity Disorder (ADHD) [44], and Alzheimer's Disease (AD) [45, 46]. For example, Lu et al. revealed that the disrupted dFC variability could distinguish patients with depressive episode from those with MDD with 83.44% classification accuracy [47]. Firouzi et al. have demonstrated that the model performance improved by up to 10% in classifying ADHD from typically developing children (TDC) using the dFC approach compared to the FC‐based classification [44]. Zhao et al. used dFC strength and a machine learning method to classify AD patients and healthy controls, finding differences in dFC strength in the left precuneus between the two groups [45].

We here capitalized on recent progress in naturalistic neuroimaging, dynamic network analyses, and neurofunctional decoding across multiple datasets to (1) determine dynamic network‐level signatures for emotional states in health individuals, and to next (2) translated these into clinical application to decode emotion‐specific diagnostic markers based on rs‐fMRI data acquired in large samples of individuals with BD (BDs) and MDD (MDDs). Happiness and sadness are core emotional dysfunctions in both MDD and BD, yet they manifest differently between the disorders [48]. MDD is primarily characterized by a persistent inability to experience positive emotions (anhedonia) and an exaggerated response to sadness [49], whereas BD involves dysregulated mood shifts, with heightened responses to both positive and negative emotions [50]. Understanding these distinctions is critical for developing emotion‐specific biomarkers, justifying our focus on happiness and sadness in this study. Our study was conducted in two stages. **(1) Stage 1: training the DEFN in healthy individuals**. A cohort of 52 healthy individuals underwent a naturalistic (movie) emotion induction paradigm using long movie clips designed to elicit happiness and sadness. Specifically, we curated a set of 12 different 10‐min movie clips (6 happiness and 6 sadness movies) and simultaneously recorded whole‐brain fMRI responses (details see Xu et al., 2023) [30]. The dFC was assessed using a whole‐brain region‐of‐interest (ROI) template comprising 400 cortical and 32 subcortical areas. Cross‐subject and cross‐episode emotion classification models were developed based on these dFC features, enabling the identification of the DEFN. DEFN is considered as a functional network signature representing emotion‐specific brain dynamics in healthy individuals. **(2) Stage 2: translating DEFN to clinical application in mood disorder**. To assess the clinical utility of the DEFN, we applied it to rs‐fMRI datasets from 286 individuals, including MDD dataset (n = 174, MDDs = 63, Healthy Controls (HCs) = 111) and BD dataset (n = 112, BDs = 61, HCs = 51). Using a nested 10‐fold cross‐validation (CV) scheme and a linear support vector machine (SVM) model, we classified MDD/BD patients from healthy participants to validate the effectiveness of the DEFN. In the present work, we aimed to robustly identify the DEFN to uncover the emotion‐specific dysfunctional patterns underlying MDD and BD symptomatology and in turn inform diagnostics and personalized interventions.

## 2 Results

**FIGURE 1:**
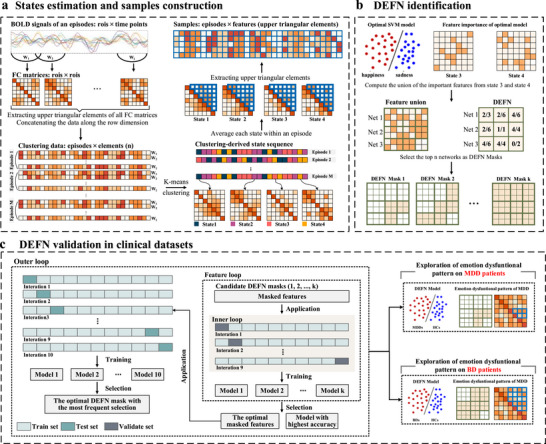
Overview of the analytical framework. (a) Procedure for dynamic state estimation using the whole‐brain template consisting of 432 regions of interest (ROIs) and sample construction in healthy individuals. (b) Identification of the DEFN in healthy individuals using the self‐acquired emotion dataset (n = 52). (c) Validation of the DEFN in clinical mood disorder datasets and exploration of emotion‐related dysfunctional connectivity patterns in the public MDD dataset (n = 174) and the self‐acquired BD dataset (n = 112).

The overall analytical framework is illustrated in Figure 1. We employ sliding time window and k‐means clustering methods to estimate 4 states that reflect dynamic brain activity during emotional episodes. For each episode, the FC matrices classified as *state_i_* are averaged as a *state_i_* matrix. The upper triangular elements of the *state_i_* matrix is extracted and vectorized into a row vector, serving as one sample for the emotional classification model input. All episodes from all participants are averaged as described above, resulting in the input samples for the model, including single‐state samples (state 1, state 2, state 3, state 4 matrices) and combined‐state samples (state 12, state 13, …, state 1234). To identify the DEFN, these samples are used to construct an optimal linear SVM model separately that distinguishes between happiness and sadness in healthy individuals, along with its stable functional features that appear in each fold of cross‐validation. The feature union derived from stable functional features appearing across states is used to calculate the network weights (i.e., DEFN) by calculating the ratio of the number of stable features to the total number of features within/between each network. The DEFN are applied to clinical datasets of mood disorders, including a publicly available dataset and a self‐acquired BD dataset to validate its effectiveness and to identify emotion‐related dysfunctional patterns within these disorders. Specifically, a nested 10‐fold CV procedure was employed to train and determine the optimal DEFN mask, which is then used to construct the classification model. Finally, the performance of the optimal model with DEFN is compared to the baseline model with whole‐brain information to assess the model efficacy, and the emotion‐related dysfunctional patterns of MDD and BD are identified based on the weights derived from the trained classification model.

### 2.1 Demographic Results

Table 1 presents the demographic and clinical characteristics of participants in the two datasets, along with the results of the statistical analyses. In the MDD dataset, compared to the HCs, MDD patients were significantly younger (t = 2.9756, p< 0.0050). There was no significant difference in sex distribution between the two groups (χ^2^ = 2.5506, p = 0.1103). As expected, MDD patients exhibited significantly higher depression severity, as measured by the Beck Depression Inventory (BDI; t = − 14.7003, p  <  0.0001). In the BD dataset, there were no significant group differences in age (t = − 1.2838, p = 0.2020), sex distribution (χ^2^ = 0.0878, p = 0.7670), or years of education (t = 1.9086, p = 0.0590). However, BD patients showed significantly serious symptom severity on clinical assessments, including the Young Mania Rating Scale (YMRS; t = − 2.0924, p  <  0.0500), BDI (t = − 2.5108, p  <  0.0500), and Beck Anxiety Inventory (BAI; t = − 3.5655, p  <  0.0050), compared to HCs. These results confirm elevated mood, depressive, and anxiety symptoms in the BD group.

**TABLE 1:** Demographic and clinical measurements of healthy individuals and patients with mood disorder.

Dataset	Characteristic	Healthy individuals	Patients	Statistics	P value
MDD dataset	Age	49.14 ± 12.29 (n = 100)	43.38 ± 10.68 (n = 58)	t = 2.9756^a)^	< 0.0050^**^
	Sex (m/f)	37/63 (n = 100)	29/29 (n = 58)	χ^2^ = 2.5506^b)^	0.1103
	BDI	8.10 ± 6.34 (n = 100)	25.71 ± 8.62 (n = 58)	t = − 14.7003^a)^	<0.0001^***^
BD dataset	Age	29.84 ± 7.13 (n = 50)	31.71 ± 7.95 (n = 59)	t = − 1.2838^a)^	0.2020
	Sex (m/f)	24/26 (n = 50)	30/29 (n = 59)	χ^2^ = 0.0878^b)^	0.7670
	Education	14.58 ± 2.30 (n = 50)	13.66 ± 2.67 (n = 59)	t = 1.9086^a)^	0.0590
	YMRS	0.16 ± 0.50 (n = 19)	0.64 ± 0.93 (n = 36)	t = − 2.0924^a)^	< 0.0500^*^
	BDI	4.53 ± 4.33 (n = 19)	9.50 ± 7.63 (n = 22)	t = − 2.5108^a)^	< 0.0500^*^
	BAI	22.7 ± 2.18 (n = 19)	28.0 ± 6.03 (n = 22)	t = − 3.5655^a)^	< 0.0010^**^

^a)^ two‐tailed two‐samples t‐test;

^b)^ two‐tailed chi‐square test. Abbreviations: BDI, Beck Depression Inventory; YMRS, Young Mania Rating Scale; BAI, Beck Anxiety Inventory.

### 2.2 Identified States and DEFN in Healthy Participants

**FIGURE 2:**
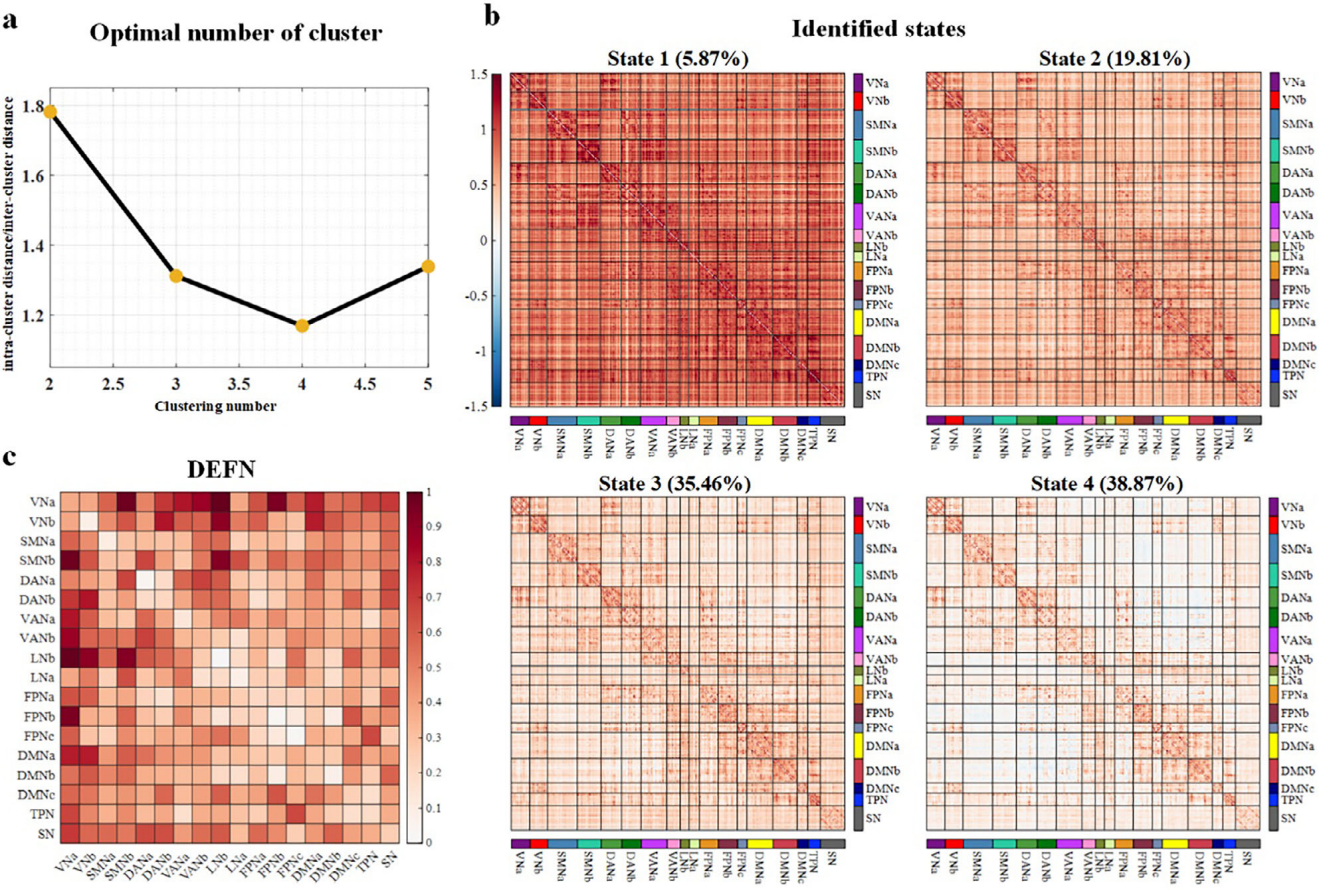
Results of estimated states and identified DEFN on healthy participants. (a) The optimal number of clusters. (b) Centroids depicted the characteristics of 4 states, with the numbers in parentheses representing the proportions of these states across all participants' time points. (c) Identified DEFN based on the optimal model. Each square indicates a network weight, with the color gradient representing the magnitude: the darker the color, the greater the network weight.

The optimal number of clusters was estimated using the elbow method and determined to be 4 clusters. The 4 recurrent states reflecting brain function during long‐term emotional experiences are depicted in Figure 2. Across all subjects and time points, the frequencies of states 1 through 4 are approximately 5.87%, 19.81%, 35.46%, and 38.87%, respectively.

**FIGURE 3:**
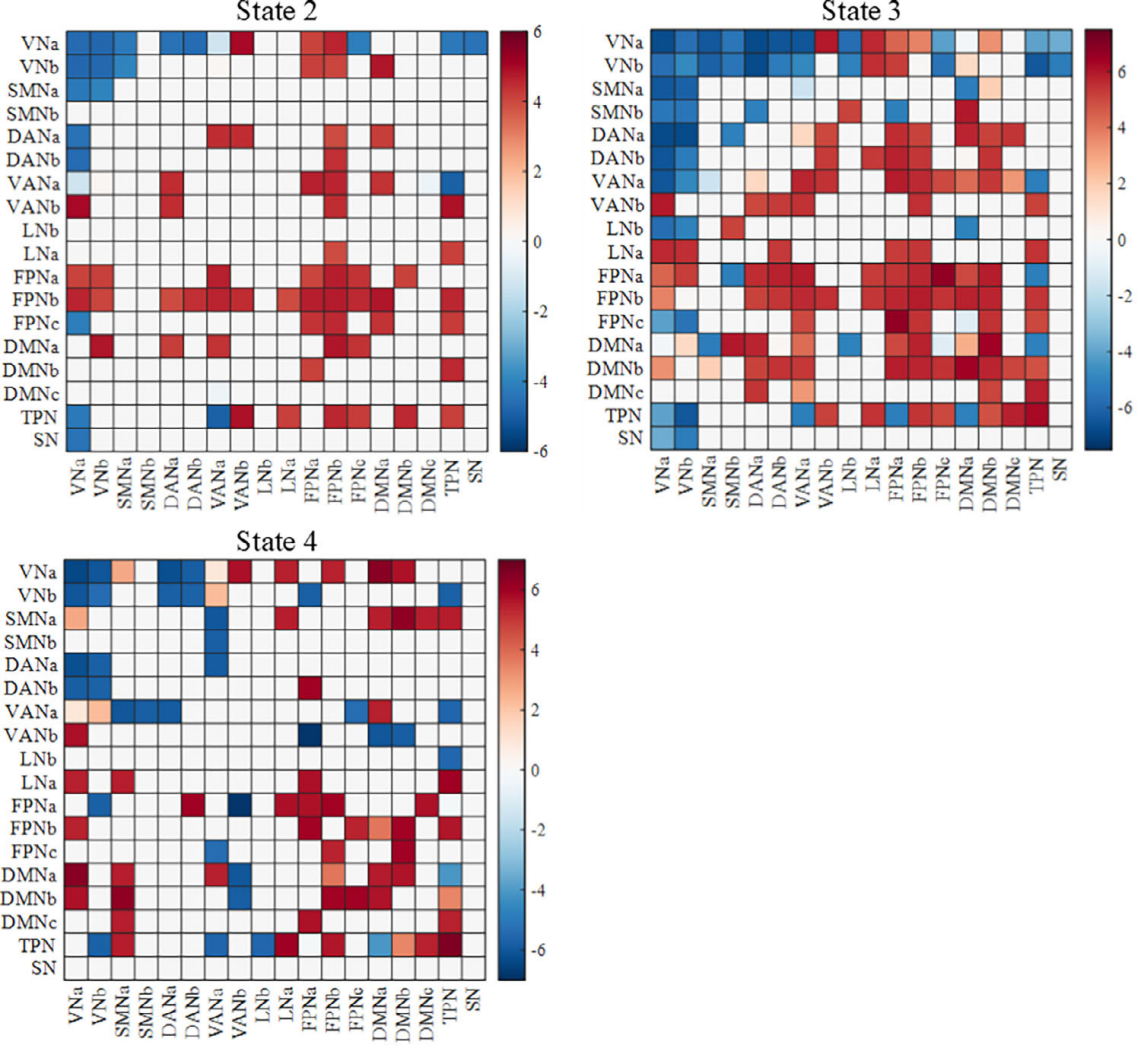
Significant differences in FC for each dynamic brain state between happy and sad movie clips. Red cells indicate connections where FC was significantly stronger during happy episodes compared to sad ones, with deeper red reflecting greater statistical difference. Blue cells indicate connections where FC was significantly stronger during sad episodes, with deeper blue reflecting greater effect size. Non‐significant connections are masked in white.

To evaluate the emotional specificity and neurobiological relevance of the 4 identified states, we examined whether each state exhibited distinct FC patterns during happy versus sad emotional experiences. The analyses (Figure 3) revealed that state 1 exhibited no significant FC alterations, suggesting it may represent a baseline or emotion‐neutral state. State 2 demonstrated limited and spatially scattered increases in connectivity, primarily between the FPN and the DMN, as well as between the DAN and VAN, indicating low emotional sensitivity and potentially reflecting a transitional or background state. In contrast, state 3 exhibited extensive increases in connectivity, particularly involving the DMN, FPN, and LN. These findings suggest that state 3 is prominently engaged during positive emotional experiences and may reflect an emotionally upregulated state with strong neurobiological specificity. State 4, on the other hand, displayed a bidirectional modulation pattern characterized by more emotion‐suppressed connections, especially within networks associated with negative emotion regulation, implying that it may correspond to a regulated or suppressed negative emotional state.

Based on the 4 identified states, feature extraction was conducted by quantifying the connectivity strength within each state and the integrated connectivity strength across all 4 states. A linear SVM model, combined with leave‐one‐subject‐out cross‐validation, was employed to decode emotional experiences of happiness and sadness. The optimal model was determined as the one yielding the highest classification accuracy and the smallest standard deviation of accuracy. From this optimal model, stable features that consistently appeared across individual cross‐validation folds were identified, and the union of these stable features across all states was obtained. Finally, the feature weights for each network were calculated based on these stable features.

The classification results (Table 2) indicate that the optimal model achieving the highest discrimination accuracy between happiness and sadness was constructed using a combination of features from state 34 (accuracy: 83.99%). Statistical analyses revealed that, except for the models constructed using features from state 3, state 4, state 23, and state 234, the model based on state 34 exhibited significantly superior performance compared to those constructed from other single or combined states. These findings suggest that, in healthy individuals, happiness and sadness can be reliably distinguished using a linear SVM model and dFC features, with a high accuracy of 83.99%. This robust performance is primarily driven by connections involving between‐network pairs associated with VNa and LNb (Figure 2).

**TABLE 2:** Performance of models in distinguishing happiness from sadness and corrected p values indicating significant differences in model performance between state 34 and the other states.

Model	Accuracy	P value	Model	Accuracy	P value
State 1	58.93 ± 31.84%	<0.0500	State 24	80.20 ± 22.53%	0.0737
State 2	73.82 ± 19.48%	<0.0500	State 34	83.99 ± 18.56%	—
State 3	81.93 ± 19.78%	0.4621	State 123	73.21 ± 24.22%	<0.0500
State 4	82.25 ± 20.82%	0.3082	State 124	76.03 ± 20.75%	<0.0500
State 12	70.16 ± 24.59%	<0.0500	State 134	71.67 ± 25.91%	<0.0500
State 13	72.54 ± 30.03%	<0.0500	State 234	84.25 ± 19.06%	0.5747
State 14	72.34 ± 24.27%	<0.0500	State 1234	75.60 ± 21.56%	<0.0500
State 23	81.93 ± 18.20%	0.3082			

### 2.3 Emotion‐Related Dysfunctional Patterns of MDD and BD

**FIGURE 4:**
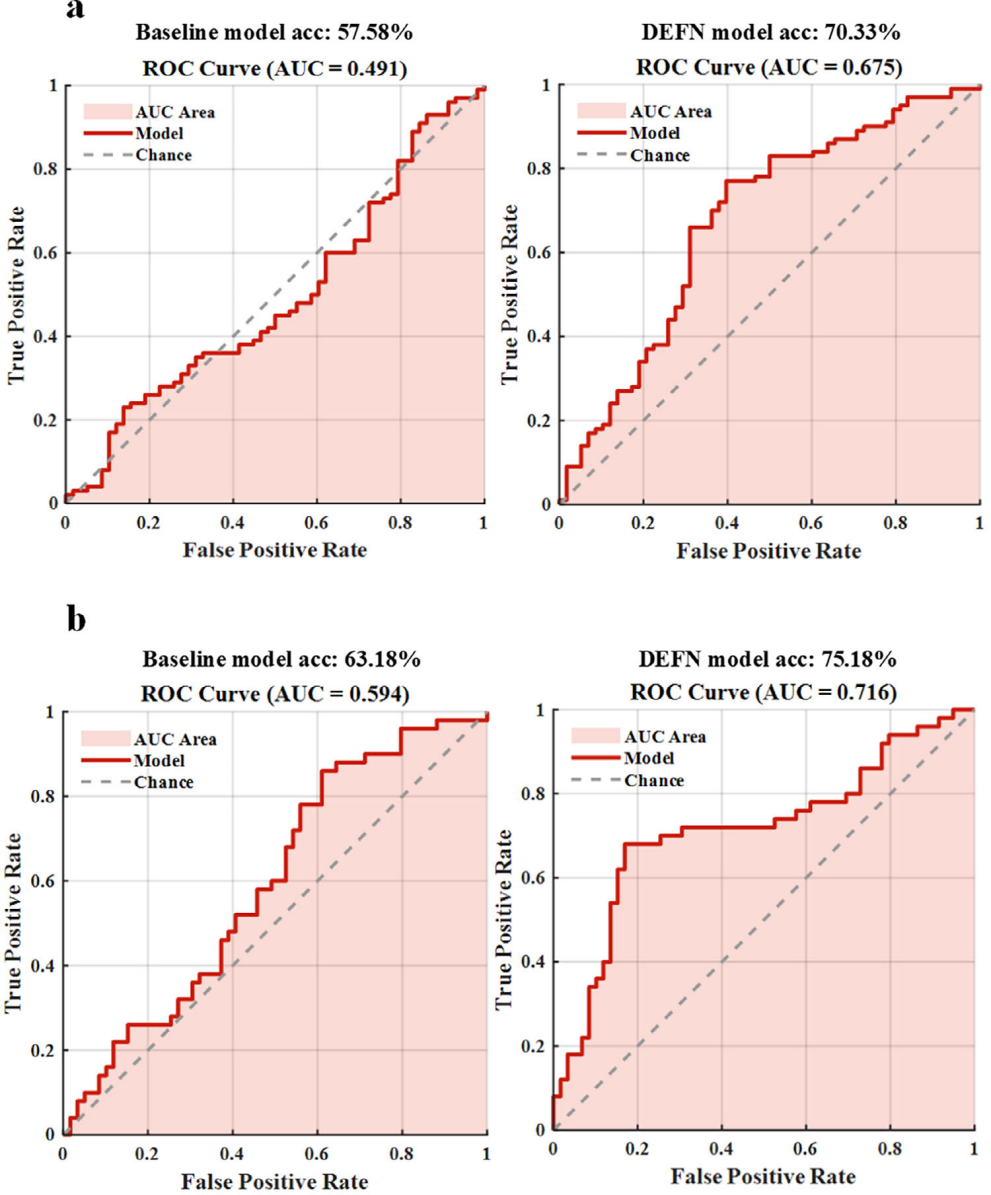
Results of model classification. (a) Receiver operating characteristic (ROC) curves and area under the curve (AUC) values of the baseline and DEFN‐based models for classifying MDD patients from healthy controls (HCs). (b) ROC curves and AUC values of the baseline and DEFN‐based models for distinguishing BD patients from HCs.

**FIGURE 5:**
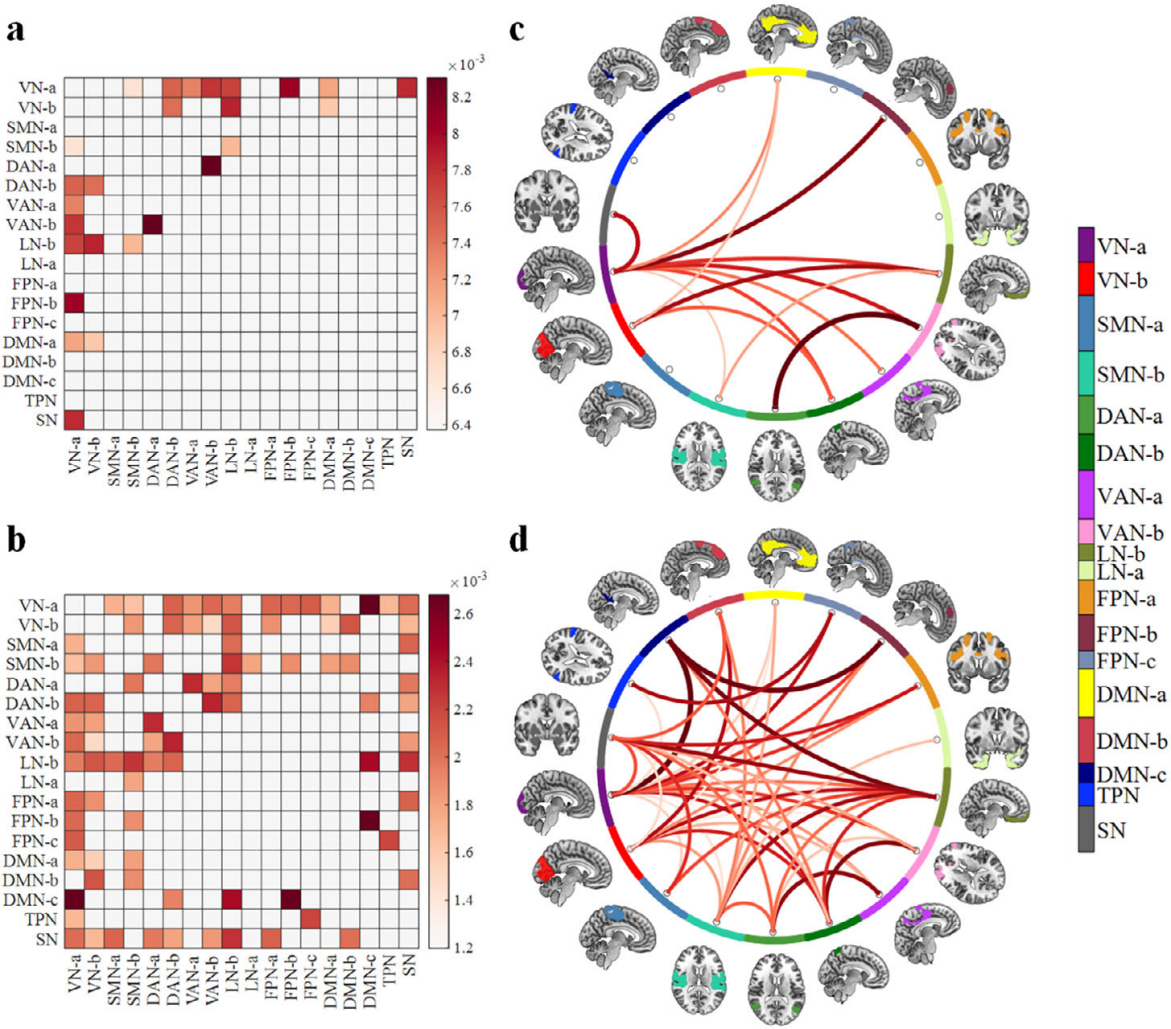
Results of emotion‐related dysfunctional patterns of MDDs and BDs. (a) Average weights of the model distinguishing MDDs from HCs. (b) Average weights of the model distinguishing BDs from HCs. The colors within the squares represent networks selected as DEFN indices, while white indicates networks that were not selected. (c) Emotion‐related dysfunctional pattern in MDDs. (d) Emotion‐related dysfunctional pattern in BDs. Each square and the thickness of the line indicate the network importance: the darker the color and the thicker the line, the greater the network importance.

To examine the neural patterns underlying emotional dysfunction in mood disorders, we focused on two clinical groups: MDD and BD. Leveraging the prior information of the DEFN derived from state 34 in healthy participants, linear SVM classifiers were constructed to discriminate patterns with MDD and BD from HCs. For each dataset, classification analyses were conducted both with and without the incorporation of the DEFN information, with the latter serving as the baseline model. McNemar's tests were used to evaluate whether the incorporation of DEFN significantly enhanced the model's classification performance. The classification results and emotion‐related dysfunctional patterns are illustrated in Figures 4 and 5.

In the MDD dataset, the model incorporating the DEFN derived from the top 13 networks achieved the best performance, with a classification accuracy of 70.33% and an area under the curve (AUC) of 0.675. Compared to the baseline model (classification accuracy: 57.58%, AUC: 0.491), the best‐performing model exhibited a significant performance improvement of 12.75% (χ^2^ = 8.0128, p  <  0.0050). Additionally, the network pairs that contribute to distinguishing MDDs from HCs (emotion‐related dysfunctional pattern of MDD) include VNa between‐network pairs (DANb, VAN, LNb, FPNb, DMNa, Subcortex Network (SN), VNb between‐network pairs (DANb, LNb, DMNa), SMNb‐LNb, and DANa‐VANb. These findings suggest that these connections may constitute an important component of the neural mechanisms underlying depression.

In the BD dataset, the model incorporating the DEFN from the top 45 networks achieved the best performance, with a classification accuracy of 75.18% and an AUC of 0.716. Compared with the baseline model (classification accuracy: 63.18%, AUC: 0.594), the optimal DEFN‐based model exhibited a significant performance improvement of 12.00% (χ^2^ = 9.8776, p  <  0.0050). The network pairs contributing to the discrimination between BDs and HCs encompass not only those identified in MDD but also additional between‐network pairs involving VNb (SMNb, VAN, FPNa, DMNb, SN), SMNb (DANa, LNa, FPNb, DMNa, DMNb), DMNc (VNa, DANb, LNb, FPNb), and SN (VNb, SMNa, DANa, DANb, VANb, LNb, FPNa, DMNb). These findings underscore both the shared and distinct patterns of neural connectivity alterations in MDD and BD, providing valuable insights into the neurobiological mechanisms underlying emotional dysregulation.

### 2.4 DEFN Consistency Across Age and Sex

**FIGURE 6:**
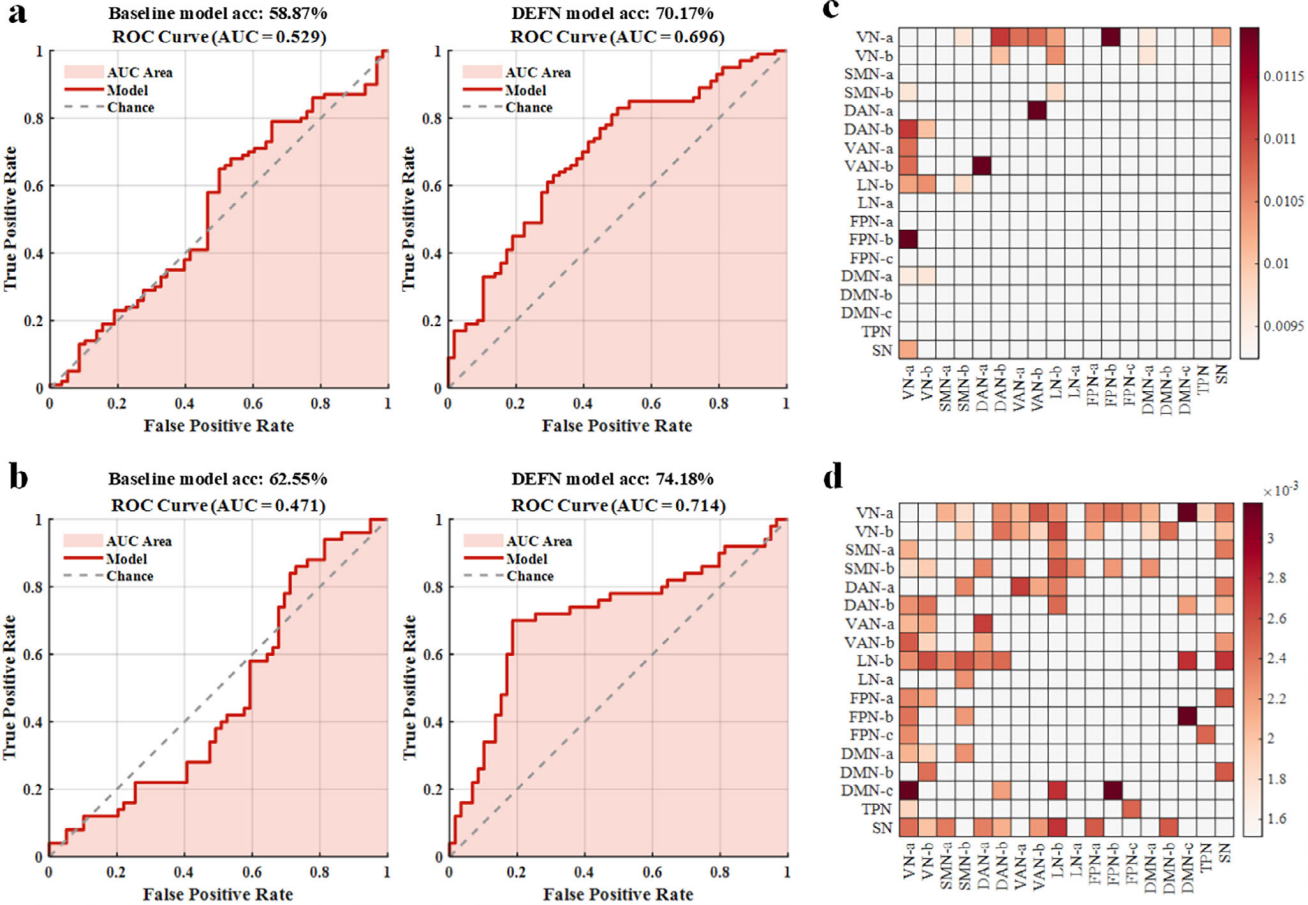
Results of classification model constructed based on static FC (sFC) features after regressing out the effects of age and sex, and emotion‐related dysfunctional patterns of MDD and BD patients. (a) ROC curve and AUC of the baseline and DEFN‐based models for classifying MDDs and HCs. (b) ROC curve and AUC of the baseline and DEFN‐based models for classifying BDs and HCs. (c) Emotion‐related dysfunctional pattern of MDDs. (d) Emotion‐related dysfunctional pattern of BDs. The colors within the squares represent networks selected as DEFN indices, while white indicates networks that were not selected. Each square and the thickness of the line indicate the network importance: the darker the color and the thicker the line, the greater the network importance.

To verify whether the DEFN mask could effectively distinguish psychiatric disorders after regressing out the effects of age and sex, and whether the optimal DEFN mask remained consistent with the results obtained without regression, we conducted a regression analysis. Specifically, age and sex were regressed out from the MDD and BD datasets, after which a nested 10‐fold cross‐validation procedure was employed to identify the optimal DEFN mask and construct the classification model. The results (Figure 6) showed that, in the MDD dataset, the optimal DEFN mask was constructed from the top 13 network pairs, achieving a classification accuracy of 70.17% (AUC = 0.696), compared to baseline model of 58.87% (AUC = 0.529). In the BD dataset, the optimal DEFN mask was constructed from the top 43 network pairs, yielding a classification accuracy of 74.18% (AUC = 0.714), compared to baseline model of 62.55% (AUC = 0.471).

## 3 Discussion

To explore the emotion‐related dysfunctional patterns in mood disorders and facilitate the development of reliable neurobiomarkers, this study employed a novel naturalistic movie paradigm to collect fMRI signals from healthy participants during sustained experiences of happiness and sadness. Through the sliding window technique combined with clustering analysis, 4 distinct brain states reflecting dynamic emotional signatures were identified. The upper triangular elements of FC matrices corresponding to these states were extracted as model inputs, and linear SVM classifiers were constructed. The optimal model achieved a classification accuracy of 83.99%, demonstrating a robust capacity to discriminate between two core emotional states: happiness and sadness. By decoding brain signals, we identified the DEFN that characterizes the neural signature associated with the core symptoms of mood disorders. To further validate the effectiveness and generalizability of DEFN, sFC features masked with DEFN information, as well as whole‐brain sFC features, were separately extracted from the MDD and BD datasets to construct classification models. Incorporating the spatiotemporal information captured by the DEFN resulted in substantial improvements in classification accuracy of 12.75% and 12.00% when using resting‐state data to differentiate MDDs and BDs from HCs. This enhancement underscores the importance of DEFN in identifying emotion‐related dysfunctional patterns in individuals with mood disorders.

### 3.1 Dynamic State‐Specific Patterns Underlying Emotional Processing

Across all dynamic states, several consistent patterns emerged in the contrast between happy and sad emotional experiences. Common differential connectivity was observed in both intra‐ and inter‐network pairs. Specifically, greater FC during happy than sad conditions was found within the FPN, DMN, and Temporal Parietal Network (TPN), as well as between VNa‐VANb, VNa‐FPNb, FPN‐DMN, and TPN‐DMN. These patterns suggest that positive emotional experiences broadly engage the higher‐order cognitive control and self‐referential systems, facilitating the integration of perceptual and regulatory processes. Conversely, greater FC during sad than happy conditions was observed within the VN and across VNa‐DAN and VANa‐TPN connections, indicating that negative emotional states may enhance the coupling between sensory processing and attentional systems, supporting increased internalized attention and perceptual vigilance under sadness.

Beyond these shared features, state‐specific patterns further reveal how the brain dynamically reorganizes emotional processing. In state 2, sad emotions were associated with stronger connectivity across VN‐SMNa, VNa‐TPN, and VNa‐SN, reflecting enhanced integration between perceptual, sensorimotor, and subcortical systems, which may underlie increased somatic awareness and affective reactivity to negative stimuli [51, 52]. In state 3, sad emotions showed greater coupling across LNb‐VN, LNb‐DMNa, VNb‐SN, SMNa‐DMNa, SMNb‐DANa, and TPN‐FPNa, suggesting a shift toward orbitofrontal‐limbic‐attentional integration, potentially reflecting cognitive‐emotional evaluation and internalized negative affect regulation [53, 54]. In contrast, happy emotions in state 3 elicited enhanced connectivity within VAN and between VANa‐DMN, implying more flexible reorientation of attention toward positive, self‐relevant stimuli [55, 56]. Finally, in state 4, sad emotions were characterized by stronger coupling among VANa‐SMN, VANb‐FPNa, and DMN, whereas happy emotions exhibited enhanced connectivity between VNa‐DMNa and SMNa‐DMN. This pattern indicates that both emotions recruit attention‐related and default mode systems but in opposite directions‐sadness toward introspection and emotional salience, and happiness toward sensory‐cognitive integration [57, 58].

Collectively, these findings demonstrate that emotional processing is supported by dynamic and state‐dependent functional architectures, rather than static network configurations. The shared patterns highlight the stable core mechanisms of emotion‐cognition interaction, whereas the state‐specific differences capture transient reorganizations reflecting distinct emotional strategies, such as self‐referential focus during sadness and adaptive attention reorientation during happiness. These dynamic connectivity signatures may thus represent fundamental neural mechanisms through which the brain flexibly adapts to fluctuating affective contexts.

### 3.2 DEFN Reflecting Naturalistic Emotional Experiences

The decoding models trained to discriminate happiness from sadness in healthy individuals indicate that high weights in DEFN are predominantly distributed across network pairs associated with VN (DAN‐b, VAN, FPN‐b, DMN‐a), DAN‐a (VAN‐b), and LN‐b (SMN‐b). This pattern is consistent with previous studies showing that emotions involve multiple cortical and subcortical regions, with distributed patterns of activity and connectivity underlying complex emotional processes [13, 14, 59, 60]. Beyond individual brain regions, emotion generation and regulation rely on interactions among distinct networks [16, 30, 61]. For instance, studies have consistently reported the involvement of the DMN in the neural representation of emotions [62]. The DMN typically includes the medial prefrontal cortex (mPFC), posterior cingulate cortex (PCC), inferior parietal lobule (IPL), hippocampus, and other memory‐related regions [62]. Xu et al. found that using FC between multiple brain regions and networks enables more accurate decoding of happiness and sadness emotional experiences compared to using FC within a single network [30]. These findings indicate that emotions are encoded within the complex patterns of brain activity and interactions.

The complexity of emotional dysregulation makes the precise diagnosis and early intervention of mood disorders challenging. One possible reason is the existence of distinct neural representations for different emotions, with various subtypes of emotional disorders exhibiting dysregulation of specific emotions. A key symptom of depression represents anhedonia, probably related to dysfunctional reward processing and alterations in the ventral striatum and associated areas [7, 8]. These regions form a network critically involved in pleasurable experiences, including the nucleus accumbens [63], the ventral pallidum [64], and limbic areas of the prefrontal cortex (PFC), particularly the orbitofrontal cortex (OFC), anterior cingulate cortex (ACC), insular cortex [65, 66], and DMN. Our study also found that these brain regions are involved in the processing of happiness, and these regions are included in the LN‐b and DMN‐a. In addition, negative emotions such as sadness have been consistently reported to be primarily processed in subcortical regions, with additional contributions from visual areas [67, 68, 69]. The decoding results of this study also revealed that the connections between the visual regions, subcortical areas, and other networks are involved in the processing and integration of negative emotions. Considering that different mood disorders primarily involve changes in happiness and sadness, this study explores the impact of the differences between the emotion of happiness and sadness on the identification of various mood disorders.

### 3.3 Superiority of the DEFN Model in Mood Disorder Classification

This study demonstrates that incorporating the DEFN model with resting‐state data leads to improved classification accuracy compared to the baseline model. Specifically, the DEFN model achieved a classification accuracy of 70.33% for identifying MDD patients, compared to 57.58% with the baseline model. Similarly, the DEFN model classified BD patients with an accuracy of 75.18%, outperforming the baseline model's 63.18%. Furthermore, to validate the specificity and robustness of the proposed DEFN, we additionally constructed a classification model based on a prior mask derived from emotion‐related ROIs reported by Morawetz et al. [70]. The sFC features within this prior mask (hereafter referred to as the Prior model) were used to decode emotional disorders from HCs. Statistical comparisons were performed to examine performance differences between the Prior model and the DEFN‐based model. As shown in Supporting Information (Appendix VI: Validation of DEFN Usefulness), the DEFN‐based model significantly outperformed the Prior model in both datasets‐MDD: 70.33% versus 59.46% (χ^2^ = 9.5211, p < 0.0010); BD: 75.18% versus 68.73% (χ^2^ = 6.7797, p < 0.0500). These findings indicate that brain features derived from naturalistic and dynamic emotional processing provide more individualized and discriminative representations than those obtained from static, meta‐analytically defined regions. Compared to other classification results based on resting‐state functional connectivity, the classification accuracy of our baseline model is similar. For instance, a study by Gallo et al. used rs‐fMRI data from the REST‐meta‐MDD (N = 2338) and PsyMRI (N = 1039) consortia, along with SVM, to classify FC features of MDDs and HCs [71]. The results showed that the model achieved an average classification accuracy of 61% in identifying MDDs from HCs, which is similar to the performance of the baseline model in our study. Another study by Dai et al. using a Transformer‐Encoder model achieved an average classification accuracy of 68.61% on a dataset consisting of 832 MDDs and 779 HCs [72]. The lower classification accuracy of our baseline model may be because we employed a more stringent 10‐fold cross‐validation method, whereas Dai's study used a more relaxed 5‐fold cross‐validation approach. Compared to other studies, our DEFN model improved accuracy by 1.72% to 11.78%, which holds significant research value for the clinical diagnosis of MDD patients [71, 72].

### 3.4 Emotion‐Related Dysfunctional Pattern of MDD

This study accurately identifies patients with MDD from HCs based on the functional activity of the visual‐attention network, visual‐prefrontal network, and visual‐subcortex network. Prior evidence has consistently shown patients with MDD and individuals with higher levels of depression exhibit aberrant connectivity across multiple emotion‐processing domains during resting‐state, including the interactions among the VN‐DMN, VN‐VAN, and VN‐PFC [40, 73, 74]. Recent neuroimaging studies further support that the visual regions are involved in emotion processing and regulation, and that abnormal interactions between the visual regions and other networks contribute to MDD [75, 76]. For example, the dorsal visual pathway involved in visuospatial processing and the anterior/posterior regions of the right temporoparietal junction, which are associated with cognitive, emotional, and social processes, exhibit disrupted resting‐state functional connectivity in MDDs [77]. A study successfully quantified 86 unmedicated MDD patients and 73 HCs by measuring the multivariate distance correlation between the dorsal attention network and the visual networks [78]. These findings suggest attenuated functional coupling between visual and attentional systems, potentially reflecting diminished attentional engagement and reduced interest in external stimuli among individuals with MDD.

Several of the identified connections involved the VN‐a, lateral prefrontal cortex (lPFC), and mPFC, suggesting that emotional dysfunctions in MDD may be related to the well‐documented roles of these regions in emotional processing and regulation. For instance, a meta‐analysis reported that MDD might be associated with abnormal FC in the PFC, ACC, temporal lobe, and basal ganglia [79]. The PFC is widely reported to be closely related to emotions and emotion regulation, including the lPFC (VAN‐b and FPN‐b) and mPFC (DMN‐a). Previous task‐based fMRI activation studies have reported insufficient activation of the lPFC in treatment‐naïve MDD patients during social cognitive processes [80, 81], which normalized after antidepressant treatment [82]. In a study using rs‐fMRI to classify HCs and MDDs, it was found that the ACC and lPFC were the most discriminative features during the classification process [83]. This indicates that the lPFC encodes emotional processing and information integration, and that functional dysregulation in these regions contributes to MDDs. The mPFC, as a key region of the DMN, is associated with internal representations of self and reward value [84]. Furthermore, FC within the DMN is negatively correlated with the degree of self‐rumination, suggesting that lower FC in the DMN is associated with higher levels of rumination, which is linked to a negative bias in emotional stimuli and impaired emotional self‐regulation [85].

Abnormal connectivity between visual regions and subcortical areas, particularly disrupted connections with the amygdala, is closely associated with impaired emotional processing and regulation in patients with MDD. Research indicates that the amygdala is involved in the preferential expression of emotions, with different emotions eliciting varying degrees of response in the amygdala. Moreover, MDD patients show a significantly higher preference for sad emotional faces compared to HCs. Following clinical pharmacological treatment, this processing preference in MDD patients reverses toward a more typical pattern [86]. A study based on transcranial direct current stimulation investigated MDDs and HCs. The results showed that negative emotions in the right amygdala and visual cortex of HCs were downregulated, whereas this was not the case for MDD patients. Additionally, the extent of downregulation was associated with the severity of the depression score [87]. The dysfunctions between the visual and subcortex may contribute to the persistent negative mood, impaired emotional regulation, and social withdrawal often observed in MDD, highlighting the role of these brain regions in both the onset and maintenance of the disorder.

### 3.5 Emotion‐Related Dysfunctional Pattern of BD

Emotion‐related dysfunctional patterns in BD encompass not only the connectivity patterns commonly observed in MDD but also additional brain networks‐primarily involving interactions among the VN, SMN‐b, DMN‐c, and SN)‐suggesting that BD may be characterized by broader and more intricate abnormalities in neural activity.

The networks that contributed most to the differentiation between BDs and HCs were the DMN‐c network pair, including VN‐a, FPN‐b, and LN‐b. Consistent with most previous studies on BD, abnormal functional connectivity within the DMN has been implicated in the pathophysiology of BD [88]. The disrupted functional connectivity between the DMN and VN‐a may reflect the tendency of BD individuals to engage in ruminative thinking when exposed to external emotion‐related stimuli. Moreover, the abnormal connectivity between the DMN and FPN‐b, as well as LN‐b (OFC), may indicate impaired regulation of negative emotions during rumination, leading BD patients to remain in a prolonged state of low mood. In addition, the abnormal connectivity between OFC and the SMN may underlie the impaired regulation of elevated mood, which could result in impulsive or excessive behaviors observed in BD patients.

Consistent with previous research, we found decreased connectivity between the SN and the visual cortex, sensory cortex, and attention networks. This may indicate that BD patients exhibit impaired attention and integration of visual and sensory‐motor information during euphoric states, leading to abnormal perception and response to external stimuli [89, 90, 91]. Research has shown that in BD patients, activation of the SN and PFC increases in response to extreme fear and mild pleasure [92]. This is consistent with our study, where enhanced connectivity between the SN and prefrontal cortex may suggest that these patients experience heightened sensitivity to positive and negative emotional stimuli, potentially contributing to increased emotional instability. A study utilizing rsFC (resting‐state functional connectivity) features to classify MDD and BD highlighted the significance of subcortical regions, particularly the caudate nucleus, in distinguishing between the two disorders. Based on the rsFC of subcortical regions, MDD and BD patients were successfully classified with an accuracy of 88.00% [93]. This suggests that subcortical regions are critical functional brain features for differentiating MDD from BD patients.

### 3.6 Limitation

Despite the promising findings, several limitations of this study should be acknowledged. First, we employed dynamic metrics such as dwell time and transition probability to decode happiness and sadness for the construction of the DEFN. However, the decoding performance based on these dynamic features did not show a clear advantage. This may be attributed to the inconsistency of the experimental paradigm across participants in this study. The differing content of the movie clips likely led to variability in the elicited brain states, resulting in suboptimal decoding performance. Future studies should consider using standardized naturalistic stimuli across all participants to ensure consistency in emotional engagement and brain state elicitation. Second, the DEFN was derived from a relatively small and homogeneous sample of healthy young adults using a single naturalistic paradigm. This may constrain the generalizability of the identified emotion‐related networks. Future studies should replicate the approach in larger and more diverse populations, potentially including variations in age, cultural background, or emotional stimuli, to enhance robustness and applicability. Third, this study is the absence of medication status information in the MDD dataset. Although all patients were diagnosed using standardized clinical interviews and exhibited significant depressive symptoms (as measured by the BDI‐II), the lack of documentation regarding psychotropic medication use (e.g., antidepressants or mood stabilizers) limits our ability to disentangle the effects of pharmacological treatment on functional connectivity. Given that certain medications have been shown to modulate brain network dynamics, the interpretation of the neuroimaging findings should be made with caution. Future studies incorporating detailed medication histories will be essential for validating and refining the observed brain connectivity patterns in clinical populations.

## 4 Methods

### 4.1 Naturalistic Emotion Dataset of Healthy Individuals

The dataset was self‐acquired. A total of 52 healthy right‐handed subjects (male/female: 26/26; age: 19 to 32 years old, 23.69 ± 2.36; with normal or corrected‐to‐normal vision) from Shenzhen University were recruited by an advertisement for this experiment. Sex information was collected via self‐report and recorded as female or male. Exclusion criteria for subject recruitment included neurological or psychiatric diagnosis, heavy alcohol consumption within the past six months, cardiovascular disease, severe visual impairment, and placement of metals in the body. All participants provided written informed consent prior to the start of the experiment. The study was approved by the Ethics Committee of the Brain Disorders and Cognitive Sciences Research Center, Shenzhen University (ethical approval number: CBDCS202011030010). The experiment was in line with the latest version of the Declaration of Helsinki. After quality control, data from one subject (female; 19 years old) was excluded due to excessive head motion (the exclusion criteria outlined in the data preprocessing section below). Details of the stimuli and experimental paradigm, as well as data acquisition procedures, are provided in the Supplementary Materials under the “Self‐acquired Healthy Dataset” section.

### 4.2 Clinic Resting‐State Dataset of MDD Patients

The MDD dataset was collected from the multi‐site, multi‐disorder resting‐state magnetic resonance image database created by Tanaka's team [94]. Tanaka's team has published 4 datasets: 1) the SRPBS Multi‐disorder Connectivity Dataset, 2) the SRPBS Multi‐disorder MRI Dataset (restricted), 3) the SRPBS Multi‐disorder MRI Dataset (unrestricted), and 4) the SRPBS Traveling Subject MRI Dataset. We employed the SRPBS Multi‐disorder MRI Dataset (unrestricted) for the subsequent analysis. The SRPBS Multi‐disorder MRI Dataset (unrestricted) consists of resting‐state functional and structural MRI images of patients with 8 types of mental disorders (MDD, autistic spectrum disorders, obsessive‐compulsive disorder, schizophrenia, pain, stroke, bipolar disorder, dysthymia, and others) as well as healthy participants. The dataset contains a total of 791 healthy participants and 619 patients from 11 sites. To eliminate the influence of different sites, we chose data from the same site with the highest number of healthy participants and MDD patients for analysis. The site with the highest number of participants had 174 individuals, including 111 healthy participants and 63 MDD patients. All participants completed the Beck Depression Inventory (BDI) assessment. After preprocessing, data containing excessive head movements were excluded, resulting in 158 participants (58 MDDs and 100 HCs) for subsequent analysis. Details of data acquisition procedures are provided in the Supplementary Materials under the “Public MDD Dataset” section.

### 4.3 Clinic Resting‐State Dataset of BD Patients

The dataset was self‐acquired. The participants in this study comprised 61 euthymic and medicated BD patients (30 males, 31 females, age: 31.75 ± 8.12 years) and 51 HCs (25 males, 26 females, age: 30.06 ± 7.23 years). All BD patients were recruited from outpatient and inpatient departments at Shenzhen Mental Health Centre, and HCs were recruited by advertisement. The procedures of this experiment were approved by the Human Research Ethics Committee of Shenzhen Mental Health Center, and all participants in this experiment signed an informed consent form. After preprocessing, data containing excessive head movements were excluded, resulting in 109 participants (59 BDs and 50 HCs) for subsequent analysis. 54 participants (19 HCs, 36BDs) completed the Young Mania Rating Scale (YMRS) assessment, while the other 42 participants (19 HCs, 22BDs) completed the Beck Depression Inventory (BDI) and the Beck Anxiety Inventory (BAI) assessments. Details of data acquisition procedures are provided in the Supplementary Materials under the “Self‐acquired BD Dataset” section.

### 4.4 Pre‐Processing of Data

All data were subjected to the following pre‐processing steps. The functional images were preprocessed using SPM [95] and DPARSF [96] in MATLAB R2023a. Given that the scanner included dummy scans to stabilize the magnetic field, the first five volumes of each time series were not discarded. However, BD data did not include dummy scans, thus the first five volumes of each time series were discarded. The structural images were first stripped of the skull and segmented into gray matter (GM), white matter (WM), and cerebrospinal fluid (CSF) based on the results of the stripped skull. Then, all the functional images of each episode were aligned with the first volume of functional images using six head‐motion parametric linear transformations, and the functional images of each subject were coregistered with the structural images. To remove the linear drift and reduce the interference of head movements and other physiological signals, nuisance covariate regression was conducted using the Friston 24‐parameter model. The functional images were then normalized to the Montreal Neurological Institute (MNI) space. A Gaussian kernel of 6 mm (FWHM) was adopted for spatial smoothing, and bandpass filters of 0.008‐0.15 in the healthy dataset and 0.01–0.1 Hz in MDD and BD datasets were conducted for eliminating the low‐frequency drift and high‐frequency noise and improving the signal‐to‐noise ratio of the BOLD signal [27, 97]. Data with excessive head motion were discarded according to the following exclusion criteria: (1) In the healthy dataset, an episode with a maximum translation of >2 mm or rotation of >2°; In MDD and BD datasets, a run with a maximum translation of >3 mm or rotation of >3°; (2) for one subject, if >2 episodes (total 6 episodes) were discarded, data from this subject would be fully excluded.

### 4.5 Brain Parcellation

We applied the Schaefer parcellation [98] covering the whole brain cortex with 400 Regions of Interest (ROIs), and the Tian atlas [99] covering the brain subcortex with 32 ROIs, to extract the BOLD time series. For each of 432 ROIs, we averaged the BOLD time series across all voxels within each ROI. Schaefer parcellation integrated both local gradient and global similarity from rest‐state and task‐state FC. According to the clearly defined coordinates of the location of structural subdivisions in the whole‐brain cortex, the parceled ROIs could be categorized into 17 networks including Visual Network (VN: VNa, VNb), SomatoMotor Network (SMN: SMNa, SMNb), Dorsal Attention Network (DAN: DANa, DANb), Ventral Attention Network (VAN: VANa, VANb), Limbic Network (LN: LNa, LNb), FrontoParietal Network (FPN: FPNa, FPNb, FPNc), Default Mode Network (DMN: DMNa, DMNb, DMNc), and Temporal Parietal Network (TPN).

Besides, consistent with previous studies [100, 101], 32 subcortical ROIs were also considered based on the recently developed Melbourne subcortical functional parcellation atlas, which was obtained based on resting‐state and task‐state functionally connectivity and was consistent with the parcellation methodology in Schaefer 400. The 32 subcortical ROIs covered 7 subcortical regions, including the hippocampus, thalamus, amygdala, caudate nucleus, putamen, and globus pallidus. All the 32 ROIs were grouped into the Subcortex Network (SN). In total, 18 networks (17 cortical networks and 1 subcortical network) were included, reflecting a broad and more fine‐grained level of organization, respectively.

### 4.6 States Estimation on Healthy Participants

For the extracted BOLD time series, the sliding window method (time window length: 30 TRs, sliding step: 2 TRs) was used to estimate the FC matrices over time. Specifically, the entire BOLD time series was split into several time series segments by a fixed time window (30 TRs) length and sliding step (2 TRs). The Pearson correlation coefficient was subsequently utilized to estimate the correlation between each pair of 432 ROIs across each time window, generating a matrix (286 ×  432 ×  432) describing the dFC for each movie. Here, 286 represents the time length of dFC, and 432 represents the number of ROIs. Additionally, Fisher's Z‐transform was applied to improve the normality of the correlation coefficients.

The k‐means clustering method was employed to identify recurrent brain states under happiness and sadness based on dFC. Before conducting the clustering analysis, we determined the optimal number of clusters using the elbow method, as it significantly influences the clustering results. In the clustering analysis, the L1 distance was employed to gauge the similarity between two dFC matrices across all participants by considering their upper triangular elements. This distance metric is recognized as an effective measure for high‐dimensional data. Specifically, the K‐means clustering was performed in two steps. In the first step, for each dFC matrix (286 ×  432 ×  432) corresponding to the movie stimuli, the upper triangular elements of each time window's FC matrix (432 ×  432) were extracted and flattened into 1D row vectors. It transforms the 3D dFC matrix into a 2D matrix of size 286 ×  93096. Subsequently, the dFC matrices from all subjects and all movie clips were concatenated along the row dimension to serve as the input for clustering analysis. The L1 distance metric was employed to randomly determine the initial clustering center‐of‐mass position, which was then repeated 100 times. The resulting outcome preserves the center‐of‐mass position of the clusters. In the second step, the k‐means clustering was carried out on the episodes of all subjects. The centroids of the first step clustering were set as the initial points, and the number of iterations for k‐means was set to 1000. Finally, all dFC matrices were clustered into 4 states according to the elbow method.

### 4.7 DEFN Identification on Healthy Participants

Based on the states, the connectivity strength within each state and combinations of connectivity strengths across different states were extracted as features to decode happiness and sadness experiences, thereby identifying DEFN from the perspective of neural activity. Here, we constructed 15 decoding models, including models built based on state 1, state 2, state 3, state 4, state 12, state 13, state 14, state 23, state 24, state 34, state 123, state 124, state 134, state 234, and state 1234. Specifically, for individual states (taking state 1 as an example) and each episode, the averaged matrix of state 1 was computed by averaging all state 1 matrices. Subsequently, the upper triangular elements of the state 1 matrix were extracted as features for decoding happiness and sadness. For feature combinations across states (taking state 12 as an example), the upper triangular elements of the averaged state 1 matrix and state 2 matrix were extracted, and these connectivity features were concatenated as features. Leave‐one‐subject‐out cross‐validation and relief feature selection methods were employed to train linear SVM models.

For the constructed decoding models, classification accuracy was employed to estimate the decoding performance of the models, and the model with the highest classification accuracy was regarded as the optimal model. Based on the optimal model (i.e., the model constructed from state 34), the intersection of all features selected across different cross‐validation folds was taken as the stable features. These features are distributed across different states and networks. Subsequently, these stable features were mapped back to their corresponding states, resulting in the feature distributions for state 3 and state 4. The union of features from state 3 and state 4 was then taken, and the feature weight for each specific network was calculated as the ratio of the number of stable features within the union to the total number of features in that network. This yielded the DEFN.

### 4.8 Exploration of Emotion‐Related Dysfunctional Patterns of MDD and BD

To investigate the neural patterns of emotion‐related dysfunctional patterns in mood disorders, we explored two types of mental disorders (MDD and BD) based on the prior information of the DEFN identified from healthy participants. First, the network weights were sorted in descending order, and the top n networks were sequentially chosen as masks (for example, selecting the top‐ranked network as the mask in the first turn, and the top two networks in the second turn), resulting in a series of DEFN masks. Subsequently, these DEFN masks were individually applied to the MDD dataset and the BD dataset to extract important features from whole‐brain static functional connectivity (sFC), building classification models that identify MDDs or BDs from HCs using a nested 10‐fold CV scheme. The nested 10‐fold CV consisted of two hierarchical loops. In the outer loop, the dataset was divided into 10 folds, with 9‐fold samples used as the training set and 1‐fold samples as the test set. For each outer training set, the candidate DEFN masks and corresponding features were selected and subsequently applied for model optimization. In the inner loop, the outer training set was again divided into 10 folds, with 9‐fold samples used as the inner training set and 1‐fold samples as the validation set. For each candidate DEFN mask, a linear SVM was trained on the inner training set and applied to the validation set, yielding *acc_inner_*. After 9 repetitions, the average of *acc_inner_* was taken as the performance of that mask *acc_feature_*. After completing all feature loops, the DEFN mask with the highest *acc_feature_* was selected as the best mask for that outer iteration. This mask was then used to train a model on the outer training set and tested on the outer test set, resulting in *acc_outer_*. After 10 outer iterations, the most frequently selected mask across folds was defined as the final optimal DEFN mask. The whole‐brain sFC features were also extracted to construct the baseline model with 10‐fold CV, to evaluate whether the DEFN‐based model achieved improved performance. The importance of each network pair in the DEFN‐based model for classifying mood disorders was quantified based on the training‐derived feature weights.

### 4.9 Validation of DEFN Consistency across Age and Sex

Given the substantial age differences between participants in the MDD and BD datasets, we sought to control for the potential effects of age and sex on the classification model. Therefore, during the construction of the classification model that distinguishes patients with mood disorders from HCs using sFC features, we performed regression analyses to remove the influence of age and sex. The detailed procedure was as follows: in the outer loop, a nested 10‐fold CV was employed to divide all samples into 9‐fold samples for training and 1‐fold samples for testing. Within the feature selection loop, specific features were selected and then entered into the inner loop, where the nine training folds were further divided into eight folds for training and 1‐fold samples for validation. Age and sex effects were regressed out using the training set, and the resulting regression coefficients were applied to the validation set for model construction. The average performance across the 9 inner folds was taken as the model performance for the selected features. After completing the feature selection loop, the optimal feature set was applied to the 9 training folds in the outer loop. Similarly, the regression coefficients and selected features obtained from the training data were applied to the test set to construct the final model.

### 4.10 Statistical Analysis

For the emotional dataset, we conducted three statistical analyses: (1) An independent‐samples t‐test was conducted to compare the subjective emotional ratings between happy (n = 147) and sad (n = 146) movie clips, in order to verify the effectiveness of emotional induction. (2) To investigate the physiological significance of each state, statistical analyses were performed on the identified states as follows. For each participant, FC matrices corresponding to *state_i_* under the happy and sad conditions were averaged separately, yielding one representative FC matrix for the happy condition and one for the sad condition. Paired t‐tests were then performed on each element of the upper triangular part of these FC matrices to examine differences between conditions. For states not present in some participants, only those participants exhibiting the given state in both conditions were included in the analysis. The resulting sample sizes for states 1–4 were 33, 50, 51, and 51 participants, respectively. The resulting p values were corrected for multiple comparisons using the false discovery rate (FDR) procedure. Finally, for each network, the mean t‐value of all significant connections was computed and taken as the representative significance measure for that network. (3) To evaluate whether the optimal model truly outperformed the other models, paired statistical tests were conducted to assess the significance of performance differences. Specifically, the lillietest function was applied to examine whether the differences in accuracy between the optimal model and each of the other models followed a normal distribution. For normally distributed data, paired t‐tests were performed, whereas for data that did not satisfy the normality assumption, Wilcoxon signed‐rank tests were used. Multiple comparisons were corrected using the FDR procedure.

For the MDD dataset, two statistical analyses were conducted: (1) Group comparisons between HCs (n = 100) and MDDs (n = 58) were performed using two‐samples t‐tests for continuous variables (i.e., age and BDI scores), and a chi‐square test for categorical variables (i.e., sex distribution). (2) McNemar tests (i.e., a paired chi‐square test) were performed to determine whether there were significant performance differences between the baseline and DEFN‐based models derived from the BD dataset.

For the BD dataset, two statistical analyses were performed: (1) Two separate subsets were analyzed based on the availability of clinical measures. For the first subset (n = 54), comprising 36 BDs and 19 HCs, group differences in YMRS scores were assessed using two‐samples t‐tests. For the second subset (n = 41), including 22 BDs and 19 HCs, group differences in BDI and BAI scores were tested using independent‐samples t‐tests. Additionally, between‐group comparisons in age and years of education for the full BD dataset (HC: n = 50; BD: n = 59) were conducted using two‐samples t‐tests, and chi‐square tests were used to examine sex distribution differences. (2) McNemar tests (i.e., a paired chi‐square test) were performed to determine whether there were significant performance differences between the baseline and DEFN‐based models constructed from the BD dataset.

All statistical tests were two‐sided unless otherwise specified. For tests requiring multiple comparisons correction we applied the FDR procedure. Statistical analyses were performed using MATLAB R2023 (Statistics and Machine Learning Toolbox), and standard built‐in functions were employed as appropriate. Effect sizes and exact p values are reported in the Results and figure legends where applicable.

## Author Contributions

S.X. and Z.L. designed the study; data were collected by S.X., T.L., L.L., and L.Z.; data analysis was performed by S.X., G.H., and Z.L.; data interpretation and manuscript drafting were contributed by S.X., B.B., and Z.L.

## Conflicts of Interest

The authors declare no conflicts of interest.

## Supporting information




**Supporting File**: advs73469‐sup‐0001‐SuppMat.docx.

## Data Availability

The data that support the findings of this study are available from the corresponding author upon reasonable request.
