# Supplementary material for: Ecologically‐Valid Emotion Signatures Enhance Mood Disorder Diagnostics
Source: Adv Sci (Weinh). 2026 Jan 5;13(13):e05524. doi: 10.1002/advs.202505524 (PMC12955986; doi:10.1002/advs.202505524)
Supplement: Supplementary file 1 — Supporting File: advs73469‐sup‐0001‐SuppMat.docx. [file ADVS-13-e05524-s001.docx]

**Supporting Information**

# Appendix Ⅰ: Consistency Validation of DEFNs

**Consistency Validation of DEFNs derived from different models with good performance**: To validate the consistency of these DEFNs across models derived from state 34, state 3, state 4, state 23, and state 234, the Pearson correlation coefficient was applied to estimate the similarity across them. The results indicate that the DEFNs constructed from the optimal models exhibit strong correlations, with the correlation between any two DEFNs being statistically significant. What’s more, all models with DEFN significantly outperformed the baseline model in the MDD dataset and BD dataset.


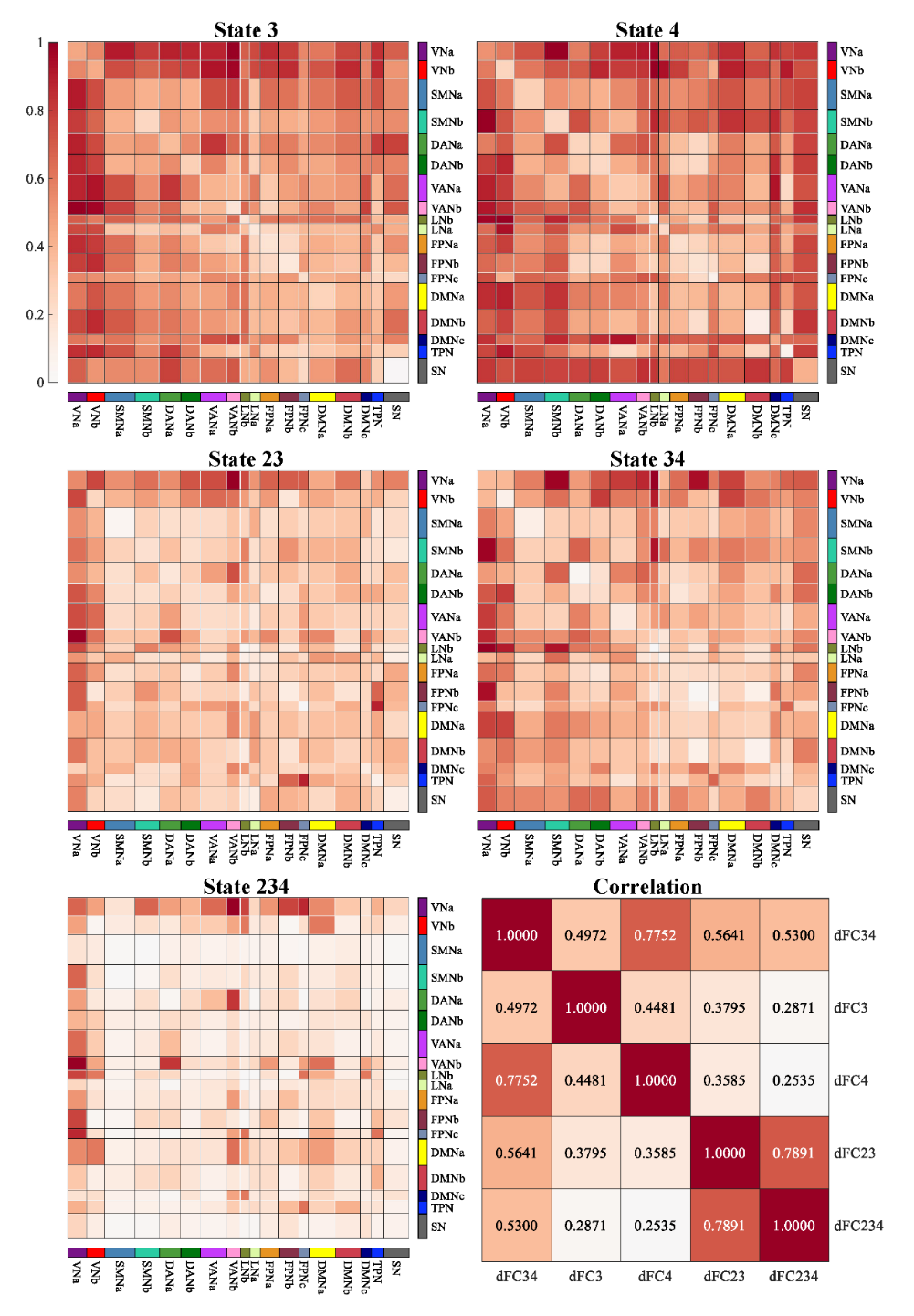


***Figure S1****. The identified DEFNs from state 3, state 4, state 23, state 234. Each network indicates the network weight, with the color gradient representing the magnitude: the darker the color, the greater the network weight. The Pearson correlation coefficient of the DEFNs obtained across state 34, state 3, state 4, state 23, and state 234.*

**Consistency Validation of DEFNs derived from different window length:** To verify the consistency of the DEFNs identified under different sliding time windows, we conducted an additional experiment. In addition to the sliding window (length = 30 s, step = 2 s) used in the main manuscript to estimate the DEFN, we further applied a window length of 60 s with a step size of 2 s and performed the same analytical procedures to identify DEFNs. Cosine similarity was then employed to quantify the similarity between the two sets of DEFNs. The results showed that the similarity between the DEFN derived from the 30-second sliding window and that derived from the 60-second sliding window was 0.7428, indicating that the DEFN is relatively robust to variations in window length, though not entirely identical.

***Figure S2.*** *Consistency results of the DEFN. a) The DEFN obtained with a sliding window of 30 s in length and a step size of 2 s. b) The DEFN obtained with a sliding window of 60 s in length and a step size of 2 s.*

# Appendix II: Data Details

## Self-acquired healthy dataset

**Stimuli:** Emotionally evocative naturalistic movie clips were employed as stimulus materials to elicit affective experiences in participants. A total of twelve 10-minute episodes were selected based on their strong and reliable ability to induce happy or sad emotional states. Specifically, the stimuli included six happy clips and six sad clips, with no content overlap among the selected episodes. The happy clips were excerpted from the films “*Mr. Popper's Penguins*”, “*Ted*”, “*The Onion Movie*”, “*Liar Liar*”, “*A Thousand Words*”, and “*Absolutely Anything*”. The sad clips were drawn from “*Miracle In Cell No.7*”, “*Prayers For Bobby*”, “*The Classic*”, “*Grave Of The Fireflies*”, “*Only The Brave*”, and “*The Last Train*”.

**Table S1.** The edited source information of the selected stimuli. The numbers 1~6 are the edited movie clips for happiness, and 7~12 are the edited movie clips for sadness.

| No. | Movie Name | Start-End | No. | Movie Name | Start-End |
| --- | --- | --- | --- | --- | --- |
| 1 | Mr. Popper's Penguins | 0:17:59-0:27:59 | 2 | Ted | 0:01:06-0:11:06 |
| 3 | The Onion Movie | 0:00:44-0:10:44 | 4 | Liar Liar | 0:38:16-0:48:16 |
| 5 | A Thousand Words | 0:22:17-0:32:17 | 6 | Absolutely Anything | 0:20:05-0:30:05 |
| 7 | Miracle In Cell No.7 | 1:20:49-1:30:49 | 8 | Prayers For Bobby | 0:39:07-0:49:07 |
| 9 | The Classic | 1:50:42-2:00:42 | 10 | Grave Of The Fireflies | 1:10:09-1:20:09 |
| 11 | Only The Brave | 1:51:43-2:01:43 | 12 | The Last Train | 1:24:33-1:34:33 |

***Figure S3****. The experimental paradigm. For each participant, six clips (three happy and three sad) were randomly selected from the full set of twelve episodes. This randomization ensured that each subject viewed a unique combination of stimuli while maintaining balanced exposure to both emotional valences.*

**Experimental paradigm:** The experimental paradigm is illustrated in Figure S3. Each participant completed six trials, corresponding to the six randomly selected movie clips. The presentation order of clips was randomized across participants to minimize potential sequence effects. Each trial comprised three phases: (1) **Baseline (30 seconds)**: Participants fixated on a white cross presented at the center of a black screen to establish a resting baseline. (2) **Movie Viewing (10 minutes):** Participants passively watched the assigned movie clip, remaining as still as possible throughout the presentation. To eliminate auditory confounds, all episodes were presented without sound but included subtitles to ensure comprehension of the narrative content. (3) **Subjective Rating:** Following each clip, participants rated the intensity and valence of their emotional experience using a five-point *happy–sad* scale ranging from –10 (very sad) to +10 (very happy). The experimental procedures were implemented and controlled using E-Prime 3.0 software.

**Data acquisition**: Brain images were obtained using a 64-channel head coil on a 3-Tesla Siemens Prisma MRI scanner. High-resolution T1-weighted structural images were acquired using a magnetization-prepared rapid acquisition gradient echo (MPRAGE) sequence with voxel resolution=1×1×1 mm^3^, repetition time (TR)=2300 ms, echo time (TE)=2.26 ms, field of view (FOV) =256×232 mm^2^, flip angle (FA)=8°. The functional images were recorded using a single gradient echo-planar imaging (EPI) sequence, with TR=1000ms, TE=30ms, FOV=192×192mm^2^, FA=90°, with a high spatial resolution of 2×2×2 mm^3^. Each volume of EPI functional images consisted of 65 slices, the total volume is 630 including 600 volumes during movie watching and 30 volumes during cross gazing. During scanning, all subjects were instructed to remain awake, keep their eyes open, and be in full engagement with the presented episodes. episodes were counter-projected on a screen and viewed through a mirror mounted on a head coil.

## Public MDD dataset

**Data acquisition**: Brain images were obtained using a 12-channel head coil on a 3-Tesla Siemens Magnetom Verio.Dot MRI scanner. The functional images were recorded using a single gradient echo-planar imaging (EPI) sequence, with TR=2500ms, TE=30ms, FOV=212×212mm^2^, FA=80°, with a spatial resolution of 3.3$\times$3.3$\times$4mm^3^. Each volume of EPI functional images consisted of 40 slices, total volume=240+4 (dummy). During scanning (10 minutes and 10 seconds dummy period), all participants were instructed to remain awake, look at the fixation point and not think about anything in particular.

## Self-acquired BD dataset

**Data acquisition:** The imaging data were performed on a Siemens 3T Trio scanner with a 12-channel head coil. Resting-state fMRI data were acquired using a standard gradient-echo EPI sequence with 31 oblique slices, TR=2000ms, TE=30ms, FOV=240×240mm^2^, FA=90°, voxel size=3$\times$3$\times$5mm^3^, total volume=246. During the whole scan, all participants were requested to keep their eyes open.

# Appendix III: Statistic results of emotion ratings

To validate the emotional effectiveness of the movie stimuli and assess whether subjective affective experience aligned with the intended emotional categories, we analyzed participants’ self-reported emotional ratings following each video segment. After each trial, participants rated their emotional experience on a 5-point scale ranging from -10 (very sad) to +10 (very happy), with 0 indicating neutral emotion. Ratings were collected for all happy and sad movie segments across all subjects in the healthy control group. We conducted a two-samples t-test comparing the emotional ratings between happy and sad video conditions. The results confirmed that emotional ratings for happy movie clips were significantly higher than those for sad movie clips (t = 28.4279, p$<$0.0001), indicating that the video materials effectively induced the intended emotional states. This behavioral validation supports the use of these segments for naturalistic emotion processing and provides further evidence that the brain states identified in the analysis are meaningfully linked to subjective affective experience.

***Figure S4****. Results of emotional ratings. Each bar represents the mean and standard error of the mean of participants’ self-reported ratings for different emotional movie clips. A significant difference was observed between ratings for happy and sad episodes.*

# Appendix Ⅳ: Comparison Between HMM and K-means dFC States

To explore whether the spatial states identified by sliding-window clustering and hidden Markov modeling (HMM) capture similar dynamic brain patterns, we implemented an HMM-Gaussian model using the HMM-MAR toolbox. The model was fit to ROI-level BOLD time series concatenated across subjects, with the number of latent states set to 4 to match the original K-means analysis. Each HMM-derived state was represented by a full covariance matrix characterizing its spatial network structure. We then computed the Pearson correlation between each HMM state’s covariance matrix and each of the 4 dFC states identified via K-means clustering on sliding-window functional connectivity. These moderate correlations indicate partial convergence in the spatial representations of brain states identified by the two methods, yet also suggest that each approach captures distinct dynamic properties due to their differing assumptions: sliding-window methods focus on connectivity patterns across temporal windows, whereas HMM models infer latent states with distinct statistical signatures directly from the raw time series. This comparison supports the notion that HMM may offer complementary insights and emphasizes the robustness of the dynamic states observed across analytical methods.

***Figure S5.*** *Results of* *4 dynamic states estimated using the HMM.*

**Table S2.** Similarity matrix between HMM-derived covariance states and K-means dFC states

|  | HMM State 1 | HMM State 2 | HMM State 3 | HMM State 4 |
| --- | --- | --- | --- | --- |
| K-means State 1 | 0.2127 | 0.2028 | 0.2076 | 0.2097 |
| K-means State 2 | 0.2510 | 0.2366 | 0.2437 | 0.2476 |
| K-means State 3 | 0.2664 | 0.2503 | 0.2583 | 0.2633 |
| K-means State 4 | 0.2634 | 0.2472 | 0.2550 | 0.2590 |

# Appendix Ⅴ: Validation of the Effectiveness of dynamic Functional Connectivity

To validate the effectiveness of dFC, we performed a direct comparison with static functional connectivity (sFC) in the task of classifying happiness versus sadness. Two separate models were constructed based on sFC and dFC, respectively. Model performance was evaluated in terms of classification accuracy and confusion matrices. The classification model based on sFC achieved an accuracy of 79.74%, whereas the model based on dFC achieved a higher accuracy of 83.99%. This indicates that incorporating the temporal dynamics of connectivity provides additional discriminative power beyond static representations. Confusion matrices for both models are shown in Figure S6, which provide a detailed overview of classification performance. As illustrated, the dFC-based model reduced misclassifications between happiness and sadness compared with the sFC-based model. These results demonstrate the added value of dynamic functional connectivity in capturing emotion-specific brain network signatures, supporting its effectiveness and superiority over static connectivity approaches.

***Figure S6.*** *Comparison of confusion matrices for the emotion classification models. a) Confusion matrix of the dFC-based model. b) Confusion matrix of the sFC-based model*

To further examine the role of temporal dynamics in classification, we performed an additional analysis based on dynamic features derived from the dFC states. After applying k-means clustering to identify four brain states, we extracted three types of dynamic features for each subject: (1) the occurrence frequency of each state, (2) the number of transitions between states, and (3) the mean dwell time of each state. These features were then used to construct classification models using a leave-one-subject-out cross-validation approach. The results indicated that the classification accuracy achieved by the dynamic features was 52.97%, which is close to the chance level. This suggests that temporal information derived from dFC states alone may not provide robust discriminative power in our paradigm. A potential explanation is that participants did not all view the exact same movie clips, resulting in individual differences in temporal dynamics that limited the effectiveness of these features in reflecting consistent emotion-related processes.

***Figure S7****. Confusion matrices of a) dFC-based model with features of states’ functional connectivity. b) dFC-based model with features of the occurrence frequency, the number of transitions between states, and the mean dwell time of each state.*

# Appendix Ⅵ: Validation of DEFN Usefulness

To ascertain that the superior performance of the optimal DEFN-based model was not solely attributable to the number of selected features, we performed a permutation-based significance analysis. Specifically, a linear SVM with 10-fold cross-validation was employed. The number of features in the optimal DEFN-based model (denoted as N) was recorded, and N features were randomly sampled from the whole-brain FC features to construct random models. For each random model, classification accuracy was computed. This procedure was repeated 5,000 times to generate an empirical distribution of accuracy values, against which the performance of the DEFN-based model was compared to assess statistical significance (i.e., p values). The results indicated that the DEFN-based model significantly outperformed the random-feature models in terms of classification accuracy (p$<$0.0001).

***Figure S******8****. Validation of the DEFN model’s effectiveness. a) Results of the permutation-based significance analysis using the top 13 DEFN features in the MDD dataset. b) Results of the permutation-based significance analysis using the top 45 DEFN features in the BD dataset.*

To assess the cross-site generalizability of DEFN, we conducted an additional validation using the MDD dataset from the University of Tokyo (UTO) site. The UTO dataset comprised 158 participants, including 96 healthy controls and 62 patients with MDD. After applying the same preprocessing pipeline to all rs-fMRI data and excluding participants with excessive head motion, 154 individuals remained (93 healthy controls and 61 MDD patients). The DEFN masks generated from the emotional dataset were applied to the MDD data from the UTO site. The optimal DEFN mask was determined using a nested 10-fold cross-validation. The results showed that the classification model constructed using the top 13 network features consistently achieved the best performance, with a classification accuracy of 72.73% and an AUC of 0.753. These findings demonstrate that the DEFN exhibits robust discriminative capability not only within the discovery site but also in an independent clinical dataset, thereby supporting its strong generalizability across different imaging centers.

***Figure S9****. Validation of the DEFN’s effectiveness in the MDD dataset from the University of Tokyo (UTO) site. a) Model performance based on receiver operating characteristic (ROC) curve metrics and the area under the curve (AUC). The red line represents the ROC curve of the model, the light red shaded area denotes the AUC, and the gray dashed line indicates the chance level. b) Emotion-related dysfunctional connectivity patterns in MDD. The colors within the squares represent networks selected as DEFN indices, whereas white squares indicate unselected networks. Each square and the thickness of its connecting line reflect network importance (the darker the color and the thicker the line, the higher the importance.*

To directly compare the performance of our DEFN-based model with previously established brain templates, we developed a prior model based on the meta-analytic emotion regulation network proposed by Morawetz’s team (Morawetz*, et al.*, 2020). Specifically, 36 regions of interest (ROIs) identified in that study-distributed across 4 functional subsystems related to emotion regulation-were selected as nodes of the prior template. We calculated the sFC between all pairs of the 36 ROIs, resulting in a feature set for classification. A linear SVM classifier was trained on these features using leave-one-out cross-validation to predict group membership on both the datasets. We performed McNemar’s chi-square tests to assess the statistical significance of classification performance differences between the DEFN-based model and the prior-based model. The results showed that the DEFN-based model significantly outperformed the prior-based model on both datasets (MDD: 70.33% vs. 59.46%, $\chi^{2}$=9.5211, p$<$0.0010; BD: 75.18% vs. 68.73%, $\chi^{2}$=6.7797, p$<$0.0500). These findings suggest that brain features derived from naturalistic, dynamic emotional processing provide more individualized and discriminative representations than static, meta-analytically defined templates.

***Figure S10****. Comparison results between the DEFN and Prior masks. a) Prior mask consisting of 36 regions of interest (ROIs); highlighted areas indicate the selected ROIs. b) Comparison of classification accuracies between the DEFN-based and prior-based models in the MDD and BD datasets. Data are presented as mean*$\pm$*standard error of the mean (SEM). ** *indicates p*$<$*0.0500, ** indicates p*$<$*0.0050.*

# Appendix Ⅶ: Validation of 4 states reproducibility

Evaluating the reproducibility of these states at the individual level is important. To evaluate the reproducibility of these 4 states at the individual level, we conducted additional analyses by identifying 4 dynamic states independently within each subject and then assessed their similarity to the group-level states using a spatial similarity analysis. The results showed that the average similarity between the subject-specific states and their corresponding group-level states was 0.65, 0.70, 0.72, and 0.75, respectively. These findings suggest a moderate to high degree of correspondence between individual and group-level brain states.

***Figure S11****. Validation results of reproducibility of states. Each dot depicts the similarity between individua-level states and group-level states.*

**References**

Morawetz, C., Riedel, M.C., Salo, T.*, et al.*, Multiple large-scale neural networks underlying emotion regulation*.* *Neuroscience & Biobehavioral Reviews*, 2020. **116**: p. 382-395.
